# Long-Term Follow-Up in Children with Anisocoria: Cocaine Test Results and Patient Outcome

**DOI:** 10.1155/2017/7575040

**Published:** 2017-12-04

**Authors:** Fabienne C. Fierz, Christina Gerth-Kahlert

**Affiliations:** ^1^Department of Ophthalmology, University Hospital Zurich, Frauenklinikstrasse 24, CH-8091 Zurich, Switzerland; ^2^Oxford Eye Hospital, Oxford University NHS Foundation Trust, Oxford OX3 9DU, UK

## Abstract

**Background:**

Evaluation of anisocoria including pharmacological testing for Horner's syndrome in the pediatric population is challenging in view of potential serious underlying disease. We describe cocaine test results, outcome of systemic investigation, and long-term follow-up in children with anisocoria.

**Methods:**

Retrospective review of medical records and phone interview of consecutive pediatric patients (<18 years old) who underwent cocaine testing from August 2007 to July 2015 at a tertiary referral centre.

**Results:**

A total of 35 patients were included with a positive, negative, or inconclusive cocaine test in 12/35, 19/35, and 4/35, respectively. Systemic investigation was performed in 11 of the patients with a positive and in 2 of the patients with an inconclusive cocaine test result. Mediastinal Hodgkin lymphoma was found in one patient with an inconclusive cocaine test result. Two other cases were presumably related to birth trauma and surgical trauma. None of the other children further developed any pathology during the follow-up period of 34.8 months (range 0–106.6).

**Conclusions:**

In most children with anisocoria and a positive cocaine test result, systemic investigation did not reveal any underlying etiology. The only malignant disease was diagnosed in a patient with a suspicion of Horner's syndrome but with an inconclusive cocaine test result in our cohort.

## 1. Introduction

Anisocoria in children is a fairly common finding. Visible anisocoria of >0.4 mm was present in 19.1% of children in a study based on measurements with an automated photoscreener in a clinical pediatric ophthalmology population, whilst anisocoria of >0.9 mm was found only in 2.3% [1].

Anisocoria with pupillary miosis on the affected side along with ptosis is the hallmark sign of Horner's syndrome. Iris heterochromia, dilation lag, anhidrosis, and harlequin sign may concurrently be present [2, 3]. Cocaine eye drops can be used to confirm the clinical diagnosis of Horner's syndrome. A resulting anisocoria value of at least 0.8 mm was effective in separating normal subjects from patients with oculosympathetic dysfunction in adults [4]. Hydroxyamphetamine drops have historically been used to distinguish preganglionic from postganglionic disease. However, as hydroxyamphetamine is usually not readily available and testing is not reliable in children due to possible transsynaptic degeneration [5], localizing tests have widely been replaced by magnetic resonance imaging of the head, neck, and upper chest [6].

Though the diagnosis of Horner's syndrome is rare in pediatric patients, potential serious underlying etiologies raise concern. Smith et al. found an incidence of pediatric Horner's syndrome of 1.42 per 100,000 patients younger than 19 years in their population-based study [7]. Lesions can be found along the oculosympathetic pathway caused by birth trauma, surgical procedures, benign and malignant neoplasia, lymphadenopathy, vascular and cerebral malformations, carotid artery thrombosis, or dissection in pediatric patients [6–15]. Neuroblastoma is the most common occult malignancy to be associated with pediatric Horner's syndrome. The prevalence of underlying mass lesions in cases of Horner's syndrome without previously identified etiologies differs from 0% to up to 21% in different studies [7–10, 16, 17]. Musarella et al. found Horner's syndrome in 14 of 405 (3.5%) patients with neuroblastoma, while it was the presenting sign in 9 (2.2%) of them [18]. As a result, there is a controversy regarding the need and extent of systemic investigation in children with Horner's syndrome [6–10].

We report results of cocaine testing and underlying etiologies as well as follow-up data in a cohort of patients younger than 18 years with anisocoria during an 8-year period.

## 2. Patients and Methods

The study was conducted at a tertiary referral centre (Department of Ophthalmology, University Hospital Zurich, Switzerland). At our institution, any use of cocaine eye drops is recorded in a separate chart. From this record, all pediatric patients (<18 years old) that underwent cocaine testing from August 2007 to July 2015 were included. Patients' medical histories, including birth history, prior surgery, age at onset and age at presentation, manifesting signs, results of cocaine testing as well as results of subsequent physical examination, catecholamine studies, and imaging studies were retrospectively reviewed. To collect follow-up data, the last follow-up visits were analyzed or parents or patients were contacted by phone regarding anisocoria and health status.

Positive cocaine tests were defined by an ophthalmologist-observed increase in anisocoria and/or resulting anisocoria of >1 mm following administration of cocaine eye drops (2.5%, 5%, or 10% depending on age) to both eyes after 30 to 60 minutes. If cocaine drops did not show appropriate dilation of either pupil, the test result was classified as inconclusive. Negative test results were defined as a decrease in anisocoria and/or resulting anisocoria of <1 mm.

The study was approved by the local ethics committee (KEK-ZH-Nr. 2016-0004) and written informed consent was obtained.

## 3. Results

A total of 38 patients younger than 18 years that underwent cocaine testing during the 8-year study period were eligible. No written informed consent was available in 3 patients, who were not included in the analysis. Median age at first notice of anisocoria was 3 months (range 0–202 months), and median age at presentation was 8 months (range 1–214 months) (Table 1). In 11 patients, anisocoria was noticed within the first month of life.

Anisocoria was confirmed in all 35 patients and was noted to be greater in dark than in bright condition in 22 patients. In the remaining cases, either difference in pupil sizes remained equal for dark and light or was not recorded. 8 cases had ptosis or possible ptosis on the side of the smaller pupil. In one patient with a negative cocaine test, ptosis was attributable to contact lens wear. Ipsilateral iris hypochromia was noted in 2 cases, and a harlequin sign was reported in 1 case.

Additional ophthalmologic findings included an anterior polar cataract on the affected side (*n* = 1), a brief episode of ocular flutter (*n* = 1) and pendular nystagmus along with horizontal gaze palsy (*n* = 1).

Cocaine test result was positive in 12 (31.4%), inconclusive in 4 (11.4%), and negative in 19 (57.1%) patients. Further evaluation by a pediatrician including physical examination, urine catecholamine testing, and imaging was initiated in 10/11 with a positive and 3/4 with an inconclusive test result as summarized in Table 2. In patient number 12 with an inconclusive test result, magnetic resonance imaging (MRI) of the head was performed for investigation of his abnormal eye movements. He was later diagnosed with compound heterozygous *ROBO3* mutation [19]. Patient number 23, who had anisocoria greater in light than in dark, had a positive cocaine test in his left eye but also showed supersensitivity to spersacarpine 0.2% in his right eye. A parasympathetic innervation deficit in his right eye was assumed and no systemic workup was performed. No further systemic investigation was initiated in patient number 34 despite an inconclusive cocaine test result as anisocoria was longstanding. Mediastinal Hodgkin's disease was found as an underlying etiology in one patient. No associated pathology was revealed in any other patient following systemic investigation. In two cases with a positive cocaine test result, birth trauma (*n* = 1) and surgical intervention (*n* = 1) were retrospectively assumed as causative factors.

Follow-up data were available in all but three patients with positive, inconclusive, or negative cocaine test results with a mean follow-up time of 34.8 months (range 0–106.6 months). No additional systemic disease related to anisocoria was reported in any of these patients.

## 4. Discussion

A majority of children in our case series was evaluated for isolated anisocoria. Many of these patients were noted to have anisocoria greater at dark than in light, which is a classical finding in Horner's syndrome. However, it is also frequently seen in physiological anisocoria [2]. Other clinical signs of possible Horner's syndrome were noted in only 8 patients. However, subtle ptosis may be difficult to assess in infants and small children and Horner's syndrome may exist without clinically detectable ptosis in up to 13% in adults [20].

All except one of the children with a positive cocaine test result in our series were referred for systemic evaluation including MRI, which did not reveal any underlying systemic pathology. A history of birth trauma or surgery was a rare explanation in our study. In other studies, the portion of Horner's syndrome related to birth trauma or previous surgery was significantly higher [8, 9]. It is possible that some of the patients with a positive cocaine test in our study had physiological anisocoria with a false-positive test result rather than true Horner's syndrome which may be an explanation for the relatively few identifiable causes found. Cocaine testing is susceptible to different sources of error such as pharmacological quality, dilution procedure, and accuracy of drop instillation. The alpha-adrenergic substance apraclonidine has increasingly been used in the diagnosis of Horner's syndrome in order to overcome these limitations in adults and children [21, 22]. However, caution is warranted as central nervous system adverse effects have been reported following diagnostic use of apraclonidine in infants [23].

More than half of patients had a negative test result (*n* = 19). No further investigation was recommended in these cases which was in retrospect the correct decision.

The only serious underlying pathology in our study was detected in a patient with an inconclusive test result. However, she did have new onset of anisocoria and ptosis suggestive of Horner's syndrome as well as concurrent weight loss which prompted further investigation and led to the diagnosis of mediastinal Hodgkin's disease. Similarly, in a recent case series of 26 infants with anisocoria, only a minority presented with an additional localizing sign such as ptosis and underlying disease was found exclusively in some of these cases [17].

The diagnostic approach in infants and children with anisocoria remains a clinical challenge. Physiological anisocoria is frequent but a diagnosis of Horner's syndrome is associated with potentially serious systemic conditions. Our results from a real-life setting highlight the need for careful clinical assessment of anisocoria and clinical features potentially associated with Horner's syndrome rather than depending on pharmacological testing alone.

## Figures and Tables

**Table 1:** Summary of patient data.

Cocaine test result	Number of patients (follow-up)	Median age at first notice (months) (range)	Congenital onset (0-1 month of life)	Median age at presentation (months) (range)	Ptosis on miotic side	Possible ptosis on miotic side	Iris heterochromia	Harlequin sign
Total	35 (32)	3.0 (0–202)	11	8.0 (1–214)	4	4	2	1
Positive	12 (11)	1.25 (0–115)	6	5.5 (1–133)	1	1	1	1
Inconclusive	4 (4)	4.5 (3–181)	0	8.0 (6–183)	2	1	0	0
Negative	19 (17)	3.0 (0–202)	5	10.0 (2–214)	1	2	1	0

**Table 2:** History and clinical findings of patients with positive and inconclusive cocaine test results.

Patient ID/gender	Age at detection (months)	Age at presentation (months)	Follow-up (months)	Birth history	Previous surgery or trauma	Additional signs in miotic eye	Additional ophthalmologic findings	Cocaine test result	Imaging	VMA/HMV	Physical examination/systemic findings	Health problems related to anisocoria at last follow-up
1/F	181	183	106.6	N/A	No	Ptosis	—	Inconclusive	CXR^a^, CT chest^b^	—	Mediastinal Hodgkin's disease	No^∗^
5/M	After birth	17	71.0	Traumatic delivery	No	—	—	Positive	—	—	Normal	No
6/M	1	6	70.3	Caesarean section	No	—	—	Positive	CXR	Normal	Normal	No
10/M	1	1	N/A	Normal	N/A	Iris hypochromia	Anterior polar cataract in miotic eye	Positive	—	Normal	Normal	N/A
11/F	After birth	2	2.0	Normal	No	—	Brief episode of ocular flutter	Positive	—	Normal	Normal	No
12/M	3	6	52.0	Vacuum	No	Possible ptosis	Pendular nystagmus, horizontal gaze palsy	Inconclusive	MRI head^c^	Normal	ROBO-3 mutation [19]	No
16/F	6	8	43.0	Normal	No	—	—	Inconclusive	—	—	Normal	No
17/F	After birth	6	29.3	Caesarean section	No	—	—	Positive	—	Normal	Normal	No
22/M	3	6	23.6	Normal	No	—	—	Positive	MRI head/neck^d^	—	Normal	No
23/M	115	133	23.6	N/A	No	—	Positive spersacarpine 0.2% test on the other eye	Positive	—	—	—	No
25/M	2	2	23.5	Normal	No	—	—	Positive	CXR, MRI head/neck/chest^e^	Normal	Normal	No
28/M	3	5	20.6	Normal	No	—	—	Positive	—	Normal	No
31/M	1	5	18.4	Normal	No	—	—	Positive	MRI total body, US abdomen	Normal	Normal	No
32/M	3	17	17.7	Normal	No	—	—	Positive	MRI head/neck/chest	Normal	Normal	No
34/F	After birth	30	14.0	Vacuum	No	Ptosis	—	Inconclusive	—	—	—	No
35/F	1	4	11.0	N/A	Closure of tracheoesophageal fistula	Harlequin sign	—	Positive	MRI head/neck/chest	Normal	Normal	No

N/A: not available; CXR: chest X-ray; CT: computed tomography; MRI: magnetic resonance imaging; US: ultrasound; VMA/HMV: vanillylmandelic acid/homovanillic acid. ^a^Mediastinal mass. ^b^Mediastinal mass with extension to the neck. ^c^Vermis abnormality. ^d^Abnormalities of gyral formation (incidental finding). ^e^MRI recommended but not performed. ^∗^Previous recurrence of Hodgkin's disease, healthy at last follow-up.
